# Supplementary material for: The interactome of intact mitochondria by cross-linking mass spectrometry provides evidence for coexisting respiratory supercomplexes
Source: Mol Cell Proteomics. 2017 Dec 8;17(2):216–32. doi: 10.1074/mcp.RA117.000470 (PMC5795388; doi:10.1074/mcp.RA117.000470)
Supplement: Supplemental Data [file supp_RA117.000470_133922_0_supp_23978_fzffwf.zip › spectra_annotation/mito_DR_spectra_annotation/488-1-72-1-10-1.pdf]

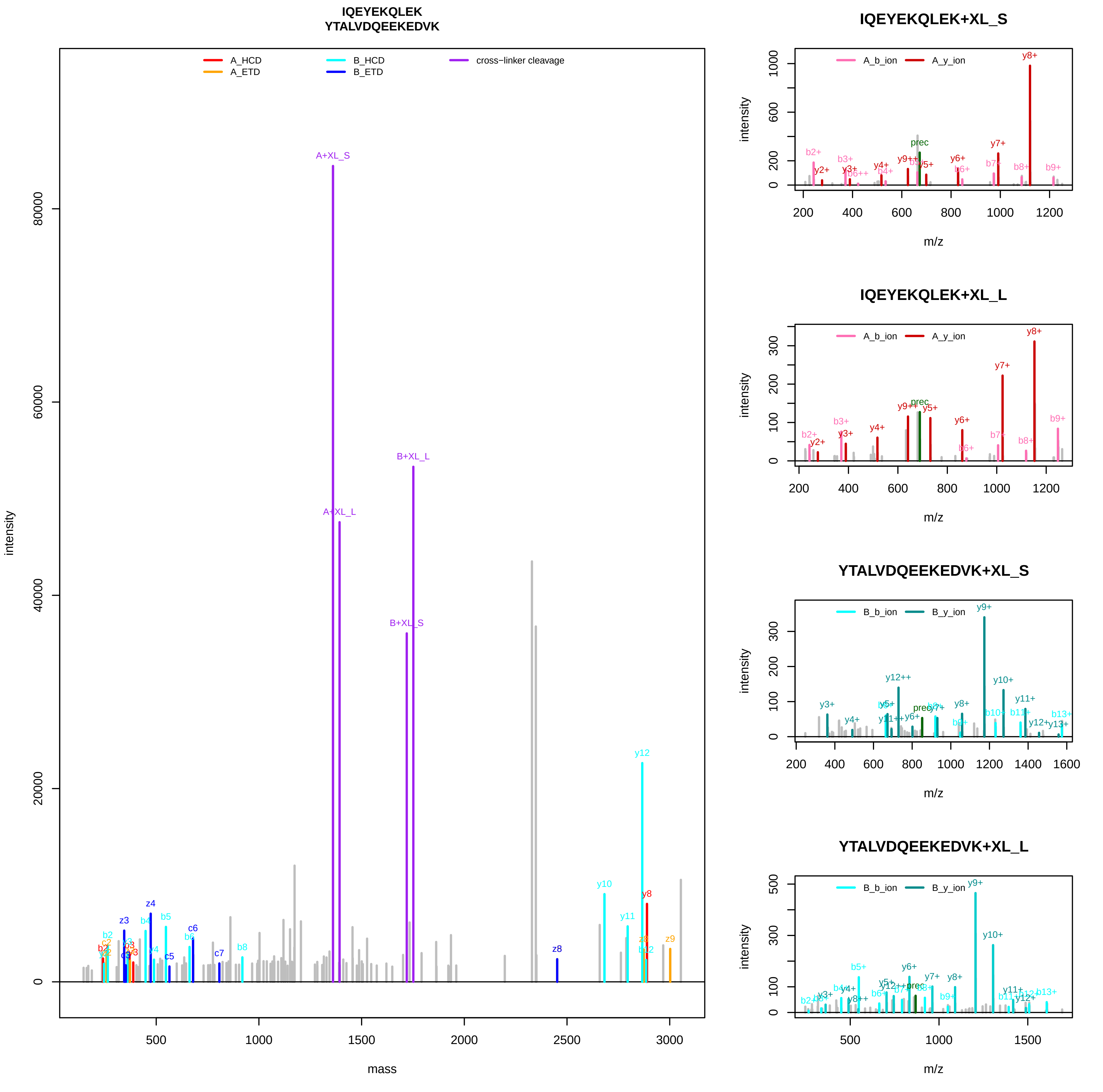

IQEYEKQLEK
YTALVDQEEKEDVK
A_HCD
A_ETD
B_HCD
B_ETD
cross−linker cleavage
A+XL_S
A+XL_L
B+XL_L
B+XL_S
intensity
mass
0
20000
40000
60000
80000
500
1000
1500
2000
2500
3000
b2
c2
y2
z3
c3
y3
b3
z4
b4
y4
b5
c5
c6
b6
c7
b8
z8
y8
y10
y11
y12
b12
z9
IQEYEKQLEK+XL_S
IQEYEKQLEK+XL_L
YTALVDQEEKEDVK+XL_S
YTALVDQEEKEDVK+XL_L
A_b_ion
A_y_ion
B_b_ion
B_y_ion
prec
m/z
y8+
y7+
y6+
y9++
y2+
y3+
y4+
y5+
b2+
b3+
b6+
b7+
b8+
b9+
y9+
y10+
y11+
y12+
y12++
y13+
b10+
b11+
b13+
